# Hepatic hemangioma in a simple liver cyst mimicking biliary cystic neoplasm

**DOI:** 10.1186/s40792-024-01908-8

**Published:** 2024-05-13

**Authors:** Ryuichi Karashima, Kensuke Yamamura, Eri Oda, Nobuyuki Ozaki, Takatoshi Ishiko, Yasunori Nagayama, Rin Yamada, Yoshihiko Komohara, Ikuro Koba, Toru Beppu

**Affiliations:** 1 Department of Surgery, Yamaga City Medical Center, Yamaga, Kumamoto 511861-0593 Japan; 2 Department of Radiology, Yamaga City Medical Center, Kumamoto, Japan; 3 Department of Cell Pathology, Graduate School of Life Sciences, Kumamoto University, Kumamoto, Japan; 4 Department of Gastroenterology, Yamaga Chuo Hospital, Kumamoto, Japan

**Keywords:** Liver, Cyst, Hemangioma, Biliary mucinous cystic neoplasm

## Abstract

**Background:**

Follow-up is recommended for an asymptomatic unilocular hepatic cystic lesion without wall-thickness and nodular components. A few liver cystic lesions represent biliary cystic neoplasms, which are difficult to differentiate from simple cysts with benign mural nodules on imaging alone.

**Case presentation:**

An 84-year-old woman with a history of simple liver cyst diagnosed one year prior was admitted for evaluation of a developed mural nodule in the cystic lesion. She had no specific symptoms and no abnormalities in blood tests except for carcinoembryonic antigen (5.0 ng/mL) and carbohydrate antigen (43.5 U/mL) levels. Contrast-enhanced computed tomography revealed a well-defined, low-attenuation lesion without a septum that had enlarged from 41 to 47 mm. No dilation of the bile duct was observed. A gradually enhancing mural nodule, 14 mm in diameter, was confirmed. MRI revealed a uniform water-intense cystic lesion with a mural nodule. This was followed by T2-enhanced imaging showing peripheral hypointensity and central hyperintensity. Enhanced ultrasonography revealed an enhanced nodule with a distinct artery within it. A needle biopsy of the wall nodule or aspiration of intracystic fluid was not performed to avoid tumor cell spillage. The possibility of a neoplastic cystic tumor could not be ruled out, so a partial hepatectomy was performed with adequate margins. Pathologically, the cystic lesion contained a black 5 mm nodule consisting of a thin, whitish fibrous wall and dilated vessels lined by CD31 and CD34 positive endothelial cells. The final diagnosis was a rare cavernous hemangioma within a simple liver cyst.

**Conclusions:**

Cavernous hemangiomas mimicking well-enhanced mural nodules can arise from simple liver cysts. In less malignant cases, laparoscopic biopsy or percutaneous targeted biopsy of the mural nodules, together with needle ablation, may be recommended to avoid unnecessary surgery.

## Background

A small subset (3–5%) of liver cystic lesions represents neoplastic precursors to cholangiocarcinoma, which included previously called biliary cystadenoma and biliary cystadenocarcinoma [1–4]. Nowadays, such biliary cystic neoplasm (BCN) includes the intraductal papillary neoplasm of the bile duct (IPNB) and biliary mucinous cystic neoplasm (BMCN) [5]. IPNB is located in the larger intrahepatic bile ducts, has macroscopically visible mucin secretion, and is accompanied by papillary mural nodules. A variety of types of neoplastic epithelium lined the cyst wall. A multiloculated cystic lesion in the subcapsular region of the liver grossly characterizes BMCN. Histologically, individual cysts vary in size and are separated by a fibrous capsule. The cysts are lined by a cuboidal to cylindrical epithelium and an underlying ovarian-like pericellular stroma. Most BMCNs show mild dysplasia but rarely severe dysplasia with associated invasive elements. Some patients have difficulty distinguishing between the two entities. The only curative treatment for such patients is early diagnosis and radical hepatectomy.

An asymptomatic unilocular cystic lesion of the liver without wall-thickness and nodular components is a candidate for follow-up [6, 7]. If diagnostic imaging reveals intracystic mural nodules with enhancement that enlarge during the course of the disease, they are strongly suspected to have malignant potential [4]. Complicated liver cysts show a variety of imaging findings reflecting intracystic hemorrhage and infection [8–11]. In such patients, the entire lesion sometimes shows high signals on the T1-weighted sequence of magnetic resonance imaging (MRI). Intracystic bleeding can cause irregularly thick walls, hyperechoic papillary structures, and enhanced mural nodules. A rare case of collagenous nodule mixed with a simple cyst and hemangioma coexistence in the liver was reported [12].

We present a rare simple liver cyst with cavernous hemangioma mimicking BCN, and diagnostic images and histopathological findings are minutely discussed.

## Case presentation

**Fig. 1:**
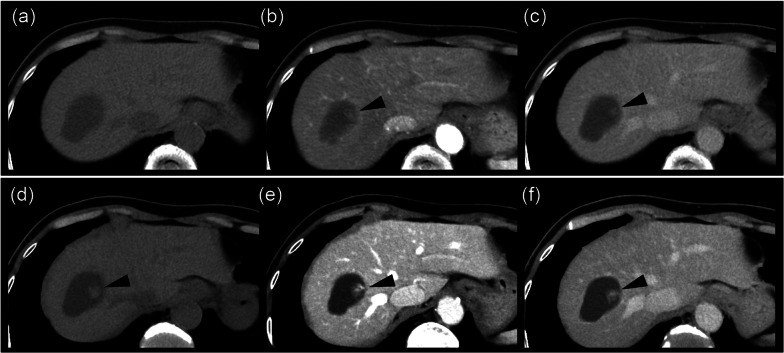
Non-contrast (**a, d**), arterial phase (**b, d**), and portal venous phase (**c, e**) computed tomography (CT) images obtained one year before surgery (**a**–**c**) and immediately before surgery (**d–f**). Over approximately one year, the irregularly shaped mural nodule (arrowheads) within the cystic lesion showed an increase in size and the degree of contrast enhancement

**Fig. 2:**
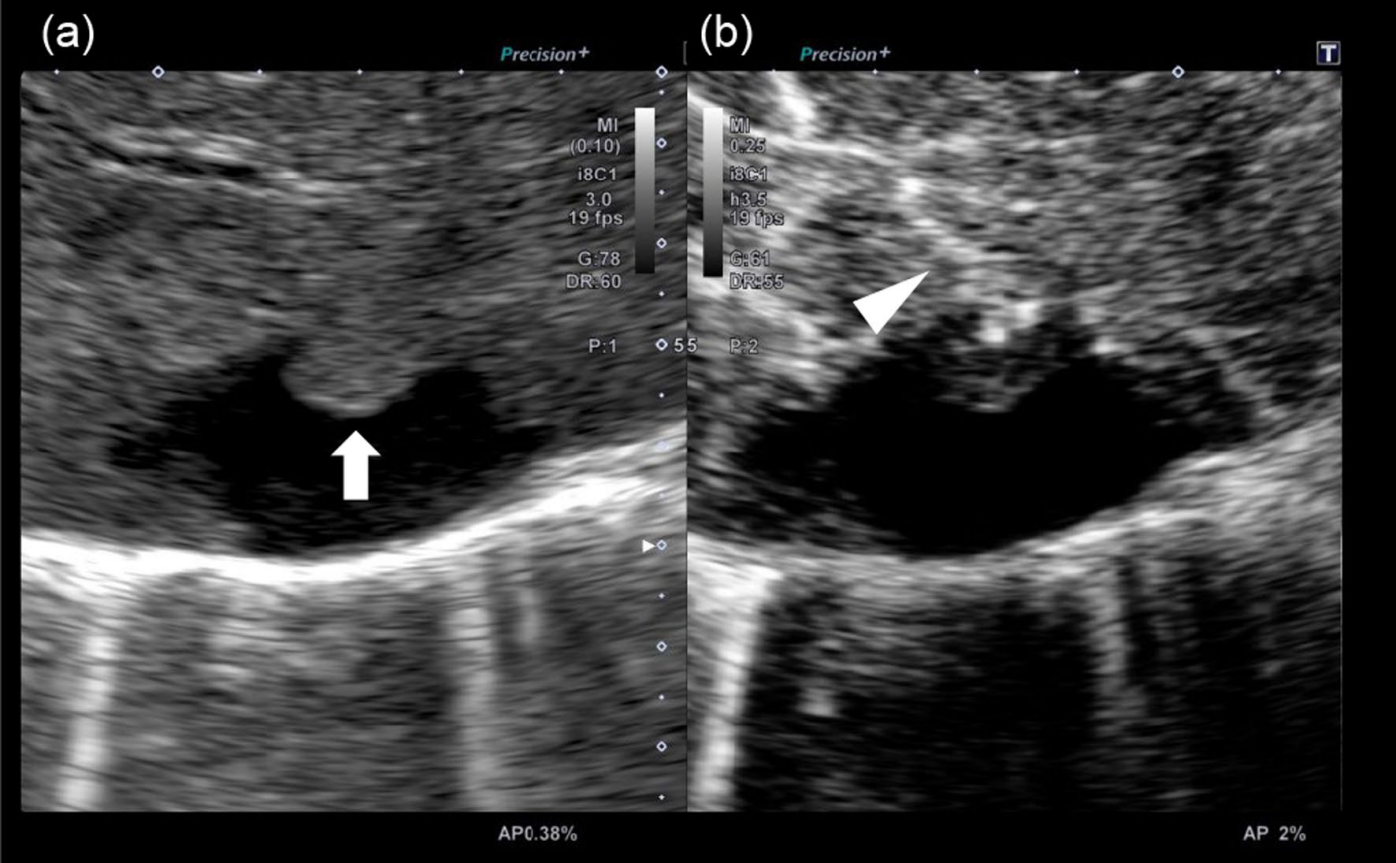
Enhanced ultrasonography. An isoechoic irregular nodule was detected in B-mode ultrasonography (**a**). A distinct inflow artery is observed toward the nodule in the enhanced ultrasonography (arrowhead) (**b**)

**Fig. 3:**
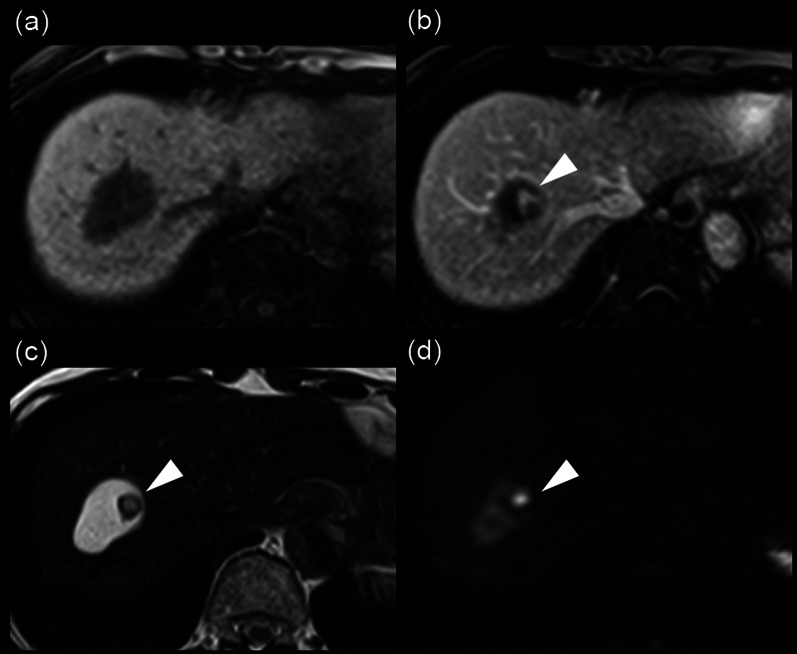
Gadolinium ethoxybenzyl diethylenetriamine pentaacetic acid-enhanced MRI. The cystic lesion is depicted as a homogeneous hypointense area, with the mural nodule not clearly visible on the non-contrast T1-weighted image (**a**). In the portal venous phase T1-weighted image, the mural nodule (arrowheads) exhibits contrast enhancement with an intensity almost comparable to that of intrahepatic vessels (**b**). T2-weighted image shows the mural nodule as an area of peripheral hypointensity and internal hyperintensity surrounded by the hyperintense cystic lesion (**c**). The mural nodule shows hyperintensity on diffusion-weighted image (**d**)

**Fig. 4:**
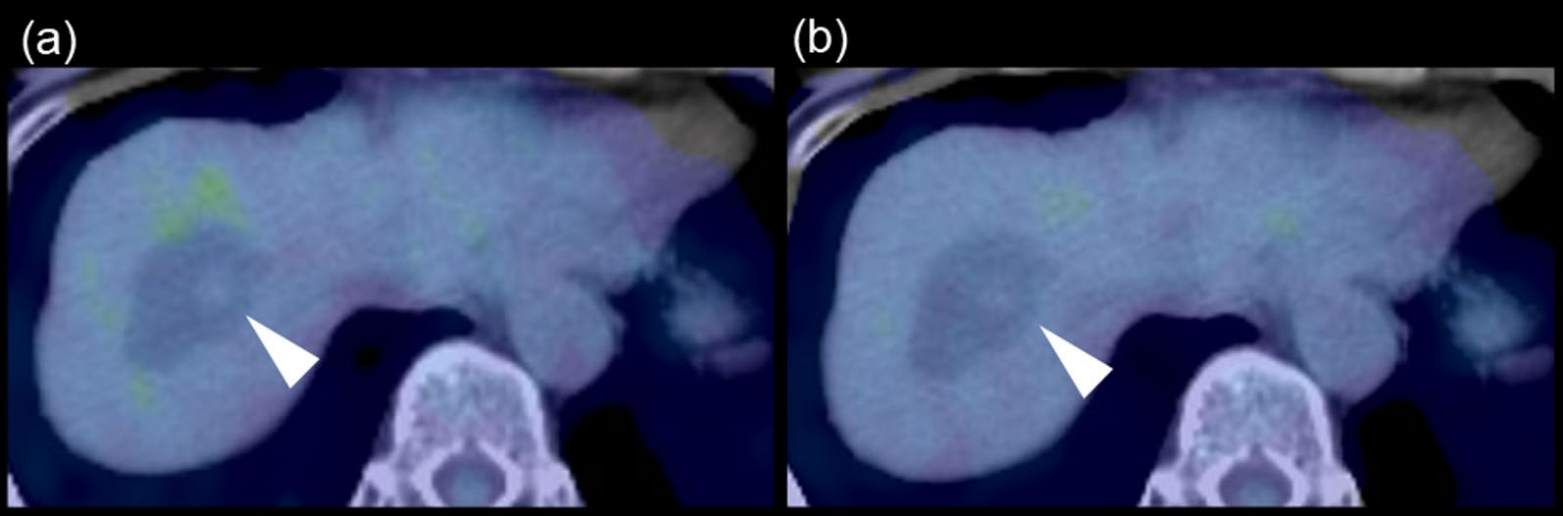
18F-fluorodeoxyglucose positron emission tomography / computed tomography images during (**a**) early and (**b**) delayed phases. No abnormal 18F-fluorodeoxyglucose uptake was observed in the nodule on either image

**Fig. 5:**
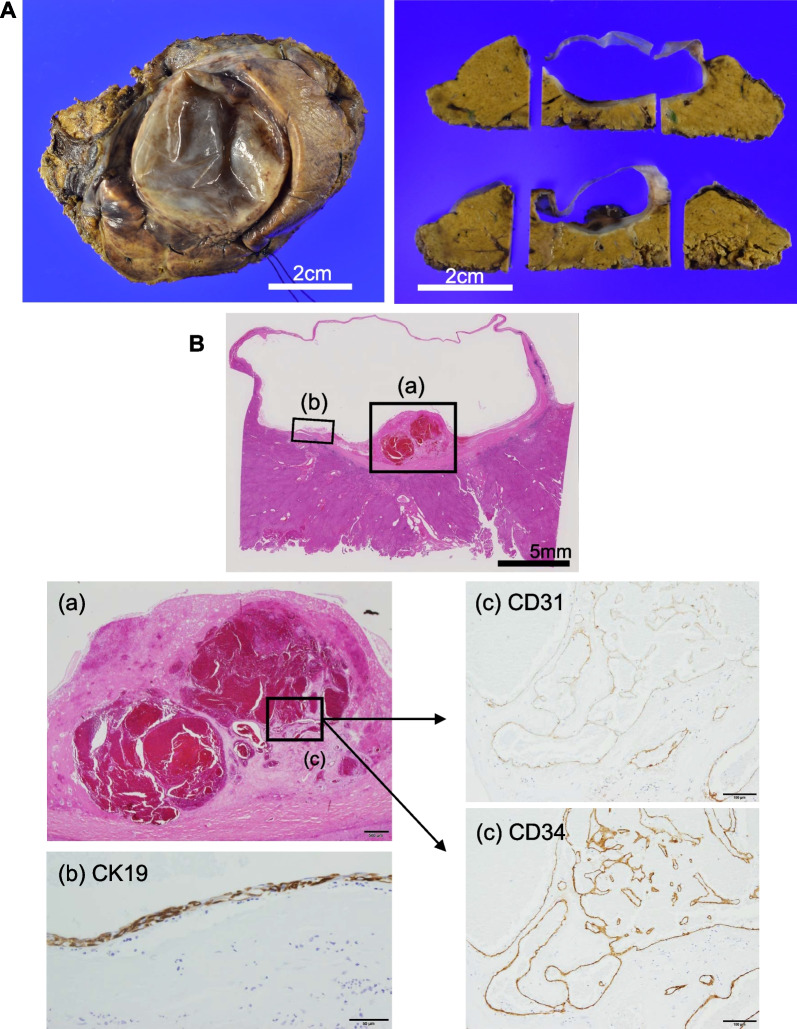
Pathological examination of liver cystic lesion. **A** Gross finding of liver cyst indicated thin whitish fibrous wall forming a 4 cm diameter cystic lesion. Within the lesion, a black-colored nodule, 5 mm in size, is observed. Scale bar; 2 cm. **B** Microscopic examination shows that the nodule is composed of dilated vessels lined with CD31- and CD34- positive endothelial cells, leading to a diagnosis of cavernous hemangioma. Cyst wall is characterized by an inner layer of cytokeratin 19 (CK19)-positive columnar epithelium and an outer layer of thin fibrous tissue

An 84-year-old woman was diagnosed with a simple liver cyst during a medical checkup. One year later, she was referred to our hospital for the evaluation of an enlarging distinct mural nodule in the liver cyst. No specific symptoms were present, and physical examinations did not reveal any abnormalities. The blood tests showed normal liver function and were negative for both hepatitis B surface antigen and hepatitis C virus antibody. However, the levels of carcinoembryonic antigen (5.0 ng/mL) and carbohydrate antigen (CA19-9; 43.5 U/mL) were slightly elevated. On contrast-enhanced computed tomography (CT), a well-defined, low-attenuation lesion without a septum was observed. The maximal diameter of the lesion had increased from 41 to 47 mm in one year (Fig. 1). No dilation of the bile duct was observed. Enhanced ultrasonography showed an enhanced nodule with a distinct artery into the nodule (Fig. 2). A 14 mm mural nodule with gradual enhancement was confirmed. MRI revealed a homogenous water intensity cystic lesion accompanied by a mural nodule, which exhibited peripheral low intensity and central high intensity on heavily T2-weighted image (Fig. 3). Positron emission tomography/computed tomography (PET/CT) did not show increased fluorodeoxyglucose activity at the mural nodule (Fig. 4). The cystic tumor was considered potentially malignant because the mural nodule was growing in size and had apparent blood flow inside, so we offered the patient two options: surgical resection or careful watching. Finally, she decided to have surgery, and partial liver resection with sufficient margins was performed. We never perform aspiration of intracystic fluid to avoid tumor cell spillage. Macroscopically, the cut surface of the resected specimen displayed a thin-walled liver cyst with a mural nodule (Fig. 5A) and included denatured liquid content. Pathological examination revealed a cystic lesion with a thin whitish fibrous wall, and a black-colored 5-mm nodule was located in the cyst wall over the normal liver tissue. The cyst wall was lined by an inner layer of cytokeratin 19-positive columnar epithelium, and the black-colored nodule was composed of dilated vessels lined with CD31- and CD34-positive endothelial cells (Fig. 5B). The final diagnosis was cavernous hemangioma in a simple liver cyst. She was discharged on the 9th day after surgery without any complications and was doing well after three months.

## Discussion

The patient had a hemangioma-like lesion one year before. Liver hemangiomas are typically caused by genetic factors, liver dysfunction, and the influence of female hormones [13]. However, it is unclear what caused the formation of the hemangioma within the simple liver cyst in the patient. There is a possibility that the hepatic hemangioma was in contact with the hepatic cyst from the beginning. The high specific findings for differentiating BCN from simple liver cyst include solitary lesions, suspicious intracystic nodular components, septal formation, septations without indentation of the cyst, upstream bile duct dilatation, and a transient hepatic attenuation difference [4, 14–17]. In contrast, the items that predict simple liver cysts include unilocular cystic lesions and septations arising only from macrolobulations.

The present case displayed a feature of BCN with diagnostic imaging modalities: a solitary lesion with an enlarging intracystic nodular component that gradually enhanced in contrast-enhanced ultrasonography, CT, and MRI. Especially in the enhanced ultrasonography with Sonazoid™, the feeding artery into the nodule was clearly defined. In contrast, the cyst wall was smooth, and enhancement of the cyst wall, as well as septations, was not observed. No dilatation of the upstream bile ducts, frequently shown in IPNB, was identified. PET/CT frequently showed strong accumulation in the marginal mural nodule of BCN, whereas fluorodeoxyglucose accumulation was not observed in benign tumors in the absence of infection [18]. The current patient showed no increased fluorodeoxyglucose activity. The differential diagnosis should include a hemorrhaging liver cyst with organized hematomas [8–11], but she had no episodes of intracystic bleeding. Calcification of the cyst wall and decreased cyst size on interval imaging is essential diagnostic clues for a hemorrhaging liver cyst with organized hematomas; however, these features were not present in the current case. We have previously reported a large liver cystic lesion accompanied by an enhanced mural nodule inside the lesion [19]. The total cystic tumor was pathologically diagnosed as degenerative hemangioma with an encapsulated hematoma. The cyst wall had no inner layer of columnar epithelium.

Some benign tumors can even exhibit slightly increased serum levels of carcinoembryonic antigen and CA19-9, and these markers can sometimes be markedly elevated in the cystic fluid [1, 15]. In the present case, the CA19-9 level in the intracystic fluid was extremely high (17,660 U/mL). Serum CA19-9 is sometimes helpful in distinguishing between malignant and benign causes; however, the CA19-9 level in the intracystic fluid is generally not practical. Aspiration of the intracystic fluid should be avoided to prevent tumor cell spillage. However, targeted biopsy of thickened walls or mural nodules, especially by laparoscopic approach, is an acceptable option to avoid unnecessary surgery for a benign tumor [20, 21]. To prevent tumor cell seeding, radiofrequency ablation of the needle tract is recommended for such situations [22].

## Conclusions

In conclusion, a patient with a simple liver cyst can develop a cavernous hemangioma mimicking a well-enhanced mural nodule. Preoperative differential diagnosis between BCN and the simple cyst is complicated only by diagnostic images; therefore, the targeted biopsy might be recommended when the possibility of malignancy is low.

## Data Availability

Not applicable.

## Abbreviations

BCN: Biliary cystic neoplasm
IPNB: Intraductal papillary neoplasm of the bile duct
BMCN: Biliary mucinous cystic neoplasm
CA19-9: Carbohydrate antigen
MRI: Magnetic resonance imaging
CT: Computed tomography
PET/CT: Positron emission tomography/computed tomography
